# Multiple effects health economic evaluation of the Ahead of The Game Study for mental health promotion in sporting club communities

**DOI:** 10.1186/s13561-021-00323-1

**Published:** 2021-08-05

**Authors:** Simon Eckermann, Nikki McCaffrey, Utsana Tonmukayakul, Christian Swann, Stewart Vella

**Affiliations:** 1 School of Health and Society, University of Wollongong, Wollongong, Australia; 2 Deakin Health Economics, Institute for Health Transformation, Deakin University, Geelong, Australia; 3 Faculty of Health, Southern Cross University, Coffs Harbour, Australia; 4 School of Psychology, University of Wollongong, Wollongong, Australia

**Keywords:** Health promotion evaluation, Mental health strategies, Multiple effect domains and dimensions, Multiple outcomes cost effectiveness analysis, Net benefit correspondence theorem

## Abstract

**Background:**

This study evaluates the Ahead Of The Game (AOTG) mental health promotion strategy for adolescent males relative to usual practice in team based sporting club community settings, allowing for joint incremental effects across 13 dimensions and 5 domains alongside intervention implementation costs.

**Methods:**

Analysis is undertaken between matched communities with difference in differences analysis of joint multiple pre-post effect changes alongside implementation costs employing radar plots in cost-disutility space. A robust bootstrapping method allowed including all observed change in effect data from 343 AOTG and 273 control arm participants across 13 effect dimensions.

**Results:**

Triangulation across joint evidence shows mean incremental effects favoured AOTG in all dimensions (10/13 significantly at 5% level) and in simple aggregation to each of five pre-specified 5 domains (each significant at < 1% level) and global measures (significant at 0.001% level), while mean AOTG implementation costs were conservatively estimated as $37.47 per participant.

**Conclusion:**

The AOTG strategy was found to represent an effective mental health promotion strategy across all domains and globally with associated significant potential for downstream health system cost savings to offset against modest implementation costs. Evaluation methods extend conventional cost-effectiveness analysis to enable robust joint presentation and triangulation under uncertainty of multiple effect dimensions alongside costs.

**Trial registration:**

ANZCTR, ACTRN12617000709347. Registered 17th May 2017.

## Background

### Evaluating health promotion in community settings for adolescent mental health: the ahead of the game (AOTG) program and study

Health promotion programs in community settings (e.g. school, workplace or sports club) adopt socio-ecological approaches considering multiple levels of influence on populations health behaviours, including intra-personal, proximal interpersonal, organisational, community, and policy levels [32] and across social, cultural, environmental, and economic determinants [24]. Jointly influencing multiple determinant and health behaviours across these multiple levels are key to such programs creating and supporting pathways and policies for meaningful community ownership, and behaviour change across target populations in community settings [5, 23, 28, 34, 35] and particularly for programs supporting mental health of adolescent male populations in community sports club settings [14, 39, 40].

Ahead of the Game (AOTG) is a comprehensive multi-level, multi-component program for adolescent males delivered through community sporting clubs and organisations [39] promoting early intervention for, and prevention of, mental illness and improved knowledge of and help seeking in use of social support networks and services. Sports clubs were chosen as an appropriate setting for the implementation of a mental health program for adolescent males because they provide access to a large proportion of the population in an engaging context. Over two-thirds of all adolescent males participate in organised sports in any given year [2], and on average spend over 8 h in organised sports each week [38]. Adolescent males also report that sports clubs are a motivating and engaging vehicle for the delivery of mental health literacy programs [37]. This is a view shared by both parents and coaches of adolescent sport participants [15, 16]. In addition, the Ahead of the Game program specifically targeted adolescent males because they are the group at highest risk of experiencing psychological difficulties, and are also the least likely to seek help [36]. For example, only 13.2% of young Australian men with a need for mental health services actually accessed the available services [36].

The aims of the program are to: (1) increase mental health literacy among adolescents and their social support systems; (2) increase help-seeking intentions and attitudes that facilitate help-seeking among adolescent male sport participants; and (3) increase resilience and factors which prevent the onset of mental health problems, including wellbeing and self-determined motivation. To address these aims, the program developed four components, two interventions for adolescents targeting mental health literacy and resilience, a parent mental health literacy intervention, and an intervention for sports club coaches which aims to help them facilitate self-determined motivation.

The AOTG study [39, 40] was designed in three phases to:

(i) develop an AOTG health promotion intervention strategy in sports club community settings that aimed to maximise club, team and participant ownership (phase one);
(ii) test and optimise implementation strategies to maximise community engagement and effectiveness while minimising implementation costs (phase two); and,
(iii) prospectively compare the optimised AOTG implementation strategy developed in the first two phases relative to usual practice at a community level evaluating across joint incremental effects and AOTG implementation costs (phase three).

The first and second phases are important in developing community health promotion strategies for complex community settings, given strategies with community ownership both enable program integration into practice and optimise potential for community level multiplier impacts over time and across community networks [7, 9, 17–20, 28, 34, 35]. Unlike individual based therapies (medications, hospital procedures etc.), community health promotion strategies where they have community ownership provide potential for community population level impacts to continue to grow in their population scope and duration of impact with integration into community practice, behaviours and lifestyle. Consequently, the community ownership and integration of health promotion strategies are key factors in assessing whether health promotion strategies and interventions in community settings are effective and cost effective at a population level in practice [7].

While optimisation of the AOTG model and the efficacy of its implementation strategy is reported elsewhere [40], this paper focuses on within study matched community level evaluation in phase three. That is, evaluation of the optimised AOTG implementation strategy relative to usual practice, with joint evaluation and triangulation of evidence across multiple effects alongside AOTG implementation costs and consideration of community level ownership, potential for broader effects and associated health system cost offsets. We use the term ‘usual practice’ instead of the term ‘usual care’ to describe the experience of participants within the control group because no explicit care regarding mental health is usually given within recreational sporting clubs. Sport participants were exposed to the standard practice of the sporting clubs to which they belonged during the study period.

## Methods

Evaluation of the AOTG study as a health promotion strategy has been designed [39] to jointly consider effect measures for intervention and control arm effects across 5 domains and 13 dimensions: global mental health (2 dimensions), resilience and preventative factors (3 dimensions), mental health literacy (4 dimensions), help seeking intentions (2 dimensions) and joint sport and mental health (2 dimensions). Variables in the global mental health domain reflect current mental health, while resilience, mental health literacy and help seeking intentions are constructs for protective factors predictive of future mental health [4, 22, 31]. The mental health sport domain captures sport specific dimensions associated with mental health. The coverage of domains, their associated dimensions measured in the AOTG study and dimension score ranges are shown in Table 1.

**Table 1:** AOTG evaluation mental health domains, variable dimensions and score ranges

Mental Health Domains	Dimension variables (variable score range)
**Global Mental Health**	Psychological distress (0–24)
	Mental health wellbeing (10–84)
**Resilience and preventative factors**	Resilience (10–50)
	Adaptive beliefs to deal with problems (3–18)
	Perceived familial support (1–7)
**Mental Health Literacy**	Depression literacy (0–13)
	Anxiety literacy (0–13)
	Social Distance (1–5)
	Confidence where to seek mental health information (1–5)
**Help seeking Intentions**	Intention to seek help - informal sources (0–7)
	Intention to seek help from formal sources (0–7)
**Mental health and sport**	Athlete engagement (1–5)
	Athlete burnout (1–5)

To estimate joint incremental effects between AOTG and usual care matched communities observed changes in pre-post effects was undertaken with difference of differences analysis [21]. Communities were matched on ARIA (Accessibility Remoteness Indicator Australia) location, geographic size, socioeconomic status, number of regions sporting clubs and participating sports. Participants were recruited from within those communities, while nested at a club and team level within their communities. Adolescent males aged 12–17 years, who participated in any community-based non-elite organised sports club from within the intervention or control communities were eligible for participation. Parents/caregivers and coaches of any adolescent participants were also invited to participate. All sports and clubs were eligible regardless of sport played. Eligible teams included those competing in the ‘Under 13′ through to ‘Under 18′ age groups. Details of the final sample can be found elsewhere [40], as can details of the recruitment and engagement strategies [39]. All participating clubs in the control community were offered a mental health literacy program following completion of the study.

To robustly consider net incremental effects and undertake health economic analysis for community based health promotion interventions such as the AOTG program requires flexible methods for triangulating evidence [41] which respectively cover and facilitate: (i) Triangulation of prospective quantitative and qualitative evidence of program community level incremental pre-post effect change between randomised or matched communities [7]; and (ii) Robust evaluation under uncertainty of joint incremental effects with multiple dimension and domain comparisons across populations, and alongside costs for incremental cost effectiveness analysis [25, 26].

Both are required for joint decision analytic coverage and comparability principles to be met and avoid expected biases for decision making that otherwise arise [6].

In relation to comparability, the AOTG study was prospectively designed to combine pre-post and matched community level evidence of AOTG effects relative to usual practice across dimensions at club, team and individual level, reflecting participant effects and community level evidence of program ownership and integration into club policies and practice [39]. Quantitatively, measuring each dimension’s score for AOTG and usual practice control arms with changes relative to baseline enables a primary mechanism to control for potential population confounders of differences between AOTG and usual practice matched communities that matching fails to control for.

In relation to coverage the net benefit correspondence theorem (NBCT) has been shown to have unique advantages in enabling flexible while robust joint multiple effect and multiple strategy cost effectiveness analysis under uncertainty. Theses advantages arise with radial properties in framing effects from a disutility (DU) bearing perspective and considering them alongside cost [6, 10–13, 25, 26]. Never-the-less previous NBCT methods in orthogonal cost-disutility (C-DU) space have been limited to 3-dimensional representation in joint consideration of 2 DU framed effects alongside costs. Hence the previous NBCT multi-dimensional cost effectiveness methods needed to be extended to allow robust joint evaluation of the AOTG studies 13 effect dimensions alongside implementation costs.

To enable robust joint comparison of 13 effect dimensions alongside costs we extend the orthogonal NBCT methods to enable joint comparison and evidence triangulation across more than 3 dimensions with use of non-orthogonal radar plots in C-DU space, while retaining the natural radial direction for comparing performance across dimensions. Joint comparison of cost and these multiple DU framed effects between AOTG and usual practice can then be robustly undertaken with radar plots in C-DU space where performance and net benefit unequivocally improve in contracting towards the origin.

In practice for the AOTG study health economics evaluation this simply involved framing observed pre-post changes in effects in AOTG and usual practice matched communities from a DU perspective as:

(i) Psychological distress;
(ii) Reduction in mental health;
(iii) Reduction in resilience;
(iv) Reduction in perceived familial support;
(v) Social Distance;
(vi) Reduction in confidence in seeking mental health information;
(vii) Depression literacy deficit;
(viii) Anxiety literacy deficit;
(ix) Reduction in confidence about where to seek mental health information;
(x) Reduction in Intention to seek help from informal sources;
(xi) Reduction in Intention to seek help from formal sources;
(xii) Reduction in athlete engagement deficit; and
(xiii) Athlete burnout.

Comparing between arms across 13 dimensions, relative disutility effect measures and cost are, as with previous NBCT methods, best presented using flexible axes for each dimension [11, 25, 26]. That is, for each strategy, each of their multiple disutility framed effects or cost dimensions can be measured relative to the lowest disutility or lowest cost strategy in comparing across strategies mean DU effect measures, or across replicates in uncertainty analysis.

These flexible axes ensure that each strategy for each dimension (DU effects measures or cost) have non-negative measures, the same common comparator and unequivocally improve when reducing, enabling key radial contraction properties in comparing relative performance improvement. Where a strategy is the best strategy for that dimension with lowest disutility or cost across strategies they will have a 0 value, while otherwise they will have an incremental DU value or cost greater than 0.

For the AOTG study analysis where variables represent pre-post change scores for the AOTG and control arm, change scores from a utility bearing perspective are simply reframed to a disutility bearing perspective as the negative of their change value from a utility bearing perspective. Hence, for example, resilience reduction for each strategy are simply the negative of resilience improvement. In comparing between strategies in DU space with flexible axes comparison for each DU axis will be relative to the strategy with lowest disutility strategy. If AOTG had a higher improvement in resilience than control or equivalently a lower incremental resilience deficit then presented with flexible DU axes incremental change in resilience deficit will be relative to that of AOTG. For AOTG incremental resilience deficit would then be relative to itself as the most effective strategy and hence have a 0 value, while the control arm has an incremental resilience deficit greater than 0 reflecting the improvement possible.

### Method for standardising absolute DU (effect) difference scores across dimensions

In interpreting incremental difference across the AOTG study Table 1 shows that the 13 effect dimensions vary considerably in their potential score ranges. Consequently, while dimension score changes can be presented as raw differences, interpreting differences in scores across dimensions or their triangulation and aggregation requires standardising for different absolute score ranges across dimensions. In comparing across the 13 dimensions incremental pre-post and case-control adjusted differences between arms, standardised incremental scores allowing for different score ranges across dimensions are calculated proportional to each variables score range. Standardised incremental differences are calculated for each dimension relative to that variables score range as:

$$ \frac{Raw\  score\ difference}{variable\ range}\times 100 $$

Hence for example if there were a 2 point improvement on:

(i) the psychological distress dimension with a variable range of 24 (0–24) this would correspond to a standardised incremental improvement of $\frac{2}{24}\times 100=8.33;$
(ii) the social distance dimension with a variable range of 4 (1–5) this would correspond to a standardised incremental improvement of $\frac{2}{4}\times 100=50.00$.

For incremental effects of any dimension this standardisation is simply interpreted as a percentage change relative to that dimensions range, while also enabling a robust basis for triangulation and simple aggregation across the 13 effect dimensions to joint consideration of domain and global levels of effect. Triangulation in aggregating from standardised dimensions to standardised domain level and from domain level to overall global effects in the base case analysis employ simple and intuitive equal weighting across dimensions to domain level effects and from domains to global effects. At a dimension or more aggregate level such simple standardisation retains advantages in representing the average percentage change and nested structure in aggregating from dimension to domain and global measures.

*A priori* an alternative form of standardisation in interpreting incremental effects across dimensions such as relative to population standard deviations in each case would in contrast create a black box to the extent they loose direct interpretability relative to instruments underlying each dimension in their aggregation to domain and or global levels as well as in comparison beyond the study population. Never-the-less directly interpretable alternative structural aggregation from across dimensions to a global levels is undertaken in sensitivity analysis, in triangulating evidence directly from a dimension level to a global level as the average of dimensions, ignoring domain level of analysis.

### AOTG resource use and costing methods

In providing and implementing the AOTG intervention, resources used for staff training and implementation time and disposables were measured at a club, team and individual participant level. Project staff time included time:

(i) for training (6 h);
(ii) attending meetings (12–15 h per staff member);
(iii) to invite a club to participate and undertake the recruitment process (2 h per club);
(iv) to recruit and deliver coach education sessions run at a club level (variable time per club);
(v) to inform and recruit team participants (variable time per team) and;
(vi) to deliver the AOTG intervention to teams (variable time per team).

Staff costs were calculated applying hourly wage rates including on-costs to each staff member’s time attributed to the club and team level where they accrued.

Staff program level costs were equally distributed across the clubs for which the staff member was responsible. Staff costs attributable to a club level included time taken to invite a club to participate and undertake the recruitment process and deliver the coach education sessions which were run at a club level. These club level staff costs were then added to disposable cost of banners and posters displayed within clubs to estimate total club costs which were in turn distributed equally between clubs participating teams. Direct team level costs included the time taken to inform and recruit team participants and to deliver the intervention to teams. Participant level costs covered the disposable merchandise package provided to each participant and was microcosted.

Total costs for each AOTG participant added the disposable per participant costs to club and team level costs, which were conservatively attributed down only to participants who provided any pre-post effect change data consistent with analysis on effects. While this is conservative in attributing AOTG costs from a team and club level down to a participant level it appropriately includes all AOTG club and team level costs in undertaking both cost effectiveness analysis and bootstrapping under uncertainty across individuals who had any pre-post change in effect data across dimensions, consistent with evaluation of effects.

### Robust bootstrapping method allowing for joint uncertainty with missing data

To enable robust analysis under uncertainty of joint incremental effects across 13 dimension (and 5 domain) effect measures with different levels of missing data across individuals by dimension, bootstrapping was undertaken on joint participant level effects and AOTG costs to support consistency and retaining covariance between effects and costs in synthesising evidence [3]. Given multiple effect measures of pre-post change with different levels of missing data and an inability to structurally undertake imputation at club, team and individual level a robust bootstrapping approach was developed which allowed inclusion of all observed effect and associated conservatively allocated AOTG implementation cost data at an individual level.

Hence, robust bootstrapping across individuals’ joint effects and cost required retaining all observed evidence while allowing for the appropriate numerator and denominator in each replicate for each variable. This was achieved separately tracking numerator and denominator sums for each of the joint 13 effect dimensions alongside intervention costs for the AOTG arm across bootstrap populations, resampled with replacement in each replicate in an inner loop for AOTG; while repeating this process 1000 times in the outer loop. The same procedure was undertaken in the control arm for the 13 effect dimensions.

Specifically, the bootstrapping method randomly resampled with replacement for each of 1000 replicates with the same number of individuals as in the study in each arm who had any change in effect data. This included 343 participants in the AOTG arm and 273 in the control arm for base case analysis, while a football only sensitivity analysis was undertaken in a somewhat more restricted population.

### Sensitivity analysis comparing AOTG vs control in football clubs alone

Base case analysis compares pre-post effects in AOTG relative to control communities over the same winter season in 2017. Clubs, their teams and participants approached over that season within these communities were predominantly football (soccer) clubs. Never-the-less 30/343 participants in the AOTG arm were from a swimming and a rugby league club while in the control arm an Australia rules football and a basketball club contributed 13/273 participants. While analysis of pre-post effects can control for baseline differences that might arise, different sports potentially have different amenity to mental health programs for adolescent males. To allow for this potential a sensitivity analysis restricting analysis to football clubs (called soccer clubs in Australia), teams and participants alone was undertaken to see whether the inclusion of a limited number of participants from other sports clubs in baseline analysis may have influenced results.

## Results

**Fig. 1:**
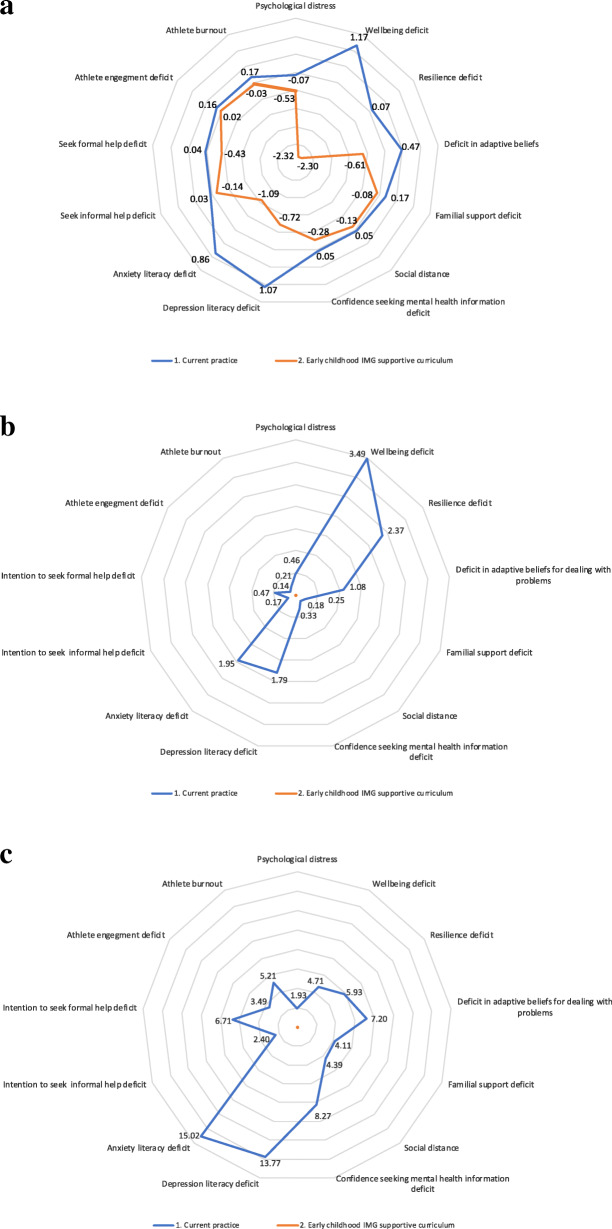
**a**: Pre-post changes in DU effects across 13 dimensions for AOTG and usual practice. **b**: Incremental DU effects with flexible axes relative to lowest DU (highest effect) strategy across 13 dimensions for AOTG and usual practice. **c**: Standardised incremental DU with flexible axes relative to lowest DU (highest effect) strategy across 13 dimensions

Pre-post DU changes across the 13 mental health dimensions for the AOTG program and usual practice arms are shown in Fig. 1a. Across strategies performance with disutility framed effects intuitively improves in moving for any dimension directly (radially) towards the origin. Hence in Fig. 1a, AOTG has lower DU (equivalently higher effect) that usual practice on each of the 13 dimensions across 5 domains.

This is more clearly seen in relative terms between arms across dimensions in Fig. 1b where incremental effects are presented with flexible axes measured relative to the lowest DU (highest effect) strategy on each dimension. Using these flexible axes the AOTG strategy is highlighted as the most effective (lowest DU strategy) on each dimension and hence remains at the origin with 0 incremental DU relative to itself the most effective strategy. The usual care program with current practice has positive incremental DU on each dimension relative to the AOTG program and hence has incremental DU relative to AOTG as the lowest DU (most effective) strategy on each of the 13 dimensions.

The absolute pre-post differences between arms presented in their natural scoring units for each of the 13 dimensions in Fig. 1b are standardised in Fig. 1c to allow interpretation of the differences in strategies as the percentage change relative to dimension score range.

**Fig. 2:**
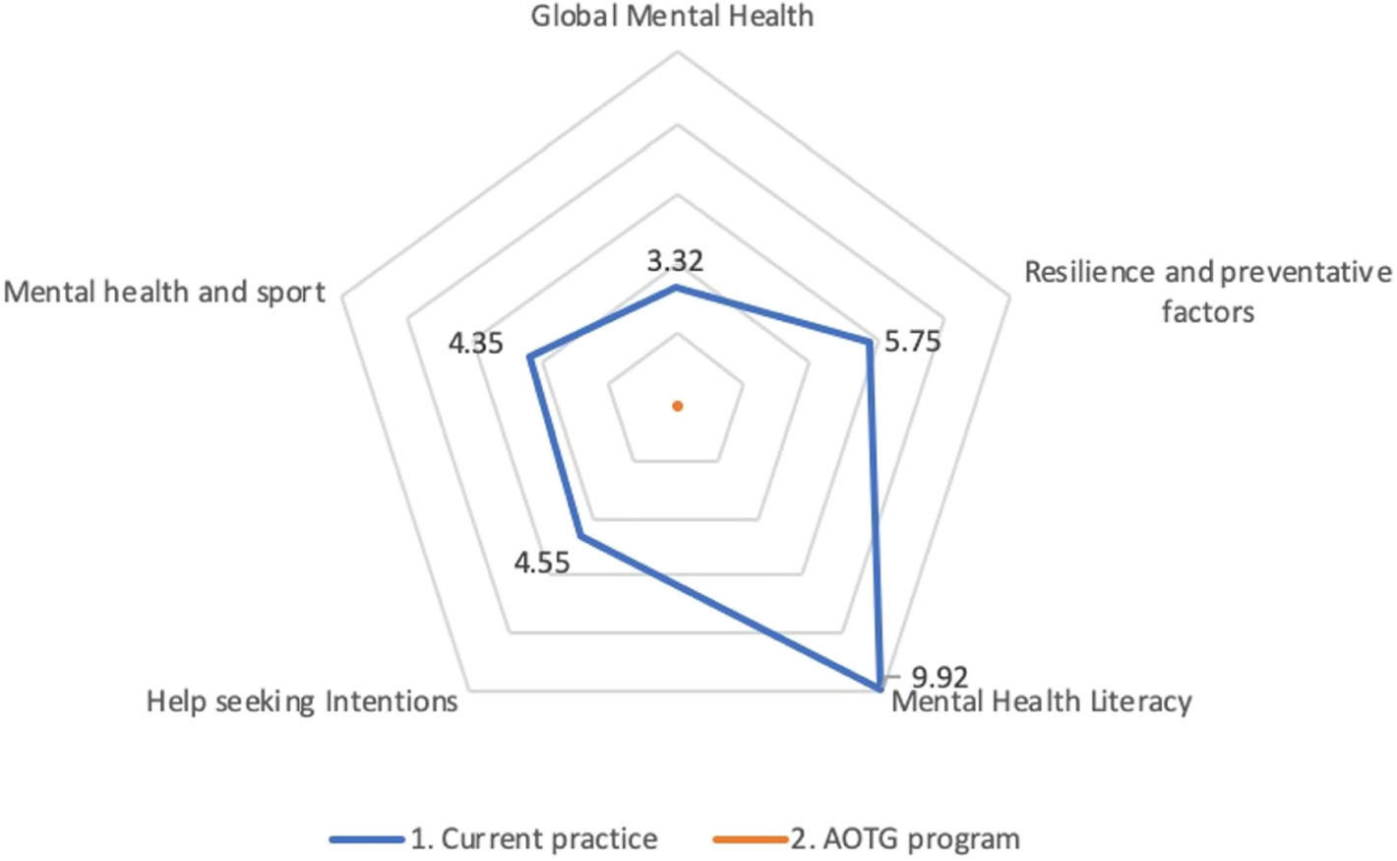
Standardised domain level incremental DU effects with flexible axes relative to lowest DU strategy for the AOTG and usual practice arms

In considering aggregated triangulation to standardised domain scores across dimensions, range standardised scores across domains as per Fig. 1c provide a common standard basis for combining dimensions to a domain level. As described in methods a priori the simplest form of aggregated domain level standardised incremental comparison is to equally weight range standardised incremental dimension scores from Fig. 1c within each domain. Standardised domain level incremental differences then represent the average percentage change across relevant standardised dimension score ranges, as shown in Fig. 2.

Figures 1 and 2c show that for each of the 13 mental health effect dimensions and their aggregation to 5 domains the AOTG study arm has lower expected disutility or equivalently higher expected effect than usual care. In standardised terms, the greatest expected improvement with AOTG is an incremental improvement of almost one tenth of the range or 9.92 points over a 100 point standardised range for mental health literacy domain, followed by 5.75 points for resilience and preventive factors and 4.55, 4.35 and 3.32 points for help seeking intentions, mental health and sport and global mental health domains, respectively.

Whether the dimensions (Fig. 1c), domains (Fig. 2) or weighted global measures from dimension level are statistically significant requires robust joint consideration across dimensions under uncertainty. Bootstrapped analysis allowing for uncertainty across the 13 effect variable dimensions jointly is summarised in Table 2 for both incremental raw scores changes and standardised score changes adjusting for different score ranges for dimensions.

**Table 2:** Mean and bootstrapped 95% CIs for raw and standardised incremental effect difference for AOTG vs control effect across 13 dimensions

Dimension	Raw score mean difference (95%CI)	Score Range	Standardised mean difference (95%CI)
Psychological distress	0.46 (− 0.13, 1.05)	0–24	1.93 (− 0.52, 4.37)
Wellbeing	3.49 (1.55, 5.41)^a^	10–84	4.71 (2.10, 7.32)^a^
Resilience	2.37 (1.21, 3.53)^a^	10–50	5.93 (3.03, 8.82)^a^
Adaptive beliefs for dealing with problems	1.08 (0.422, 1.74)^a^	3–18	7.20 (2.79, 11.61)^a^
Familial support	0.25 (0.03, 0.46)^a^	1–7	4.11 (0.57, 7.65)^a^
Social distance	0.18 (0.03, 0.32)^a^	1–5	4.39 (0.75, 8.03)^a^
Confidence seeking mental health information	0.26 (0.07, 0.49)^a^	1–5	6.48 (1.76, 11.21)^a^
Depression literacy	1.79 (1.27, 2.31)^a^	0–13	13.77 (9.75, 17.80)^a^
Anxiety literacy	1.95 (1.39, 2.51)^a^	0–13	15.02 (10.70, 19.33)^a^
Intention to seek informal help	0.17 (−0.05, 039)	0–7	2.40 (−0.71, 5.50)
Intention to seek formal help	0.47 (0.19, 0.75)^a^	0–7	6.71 (2.66, 10.76)^a^
Athlete engagement	0.14 (−0.04, 0.32)	1–5	3.49 (−0.90, 7.89)
Athlete burnout	0.21 (0.05, 0.37)^a^	1–5	5.21 (1.23, 9.19)^a^

^a^statistically significant at 5% level type I error

Table 2 shows that at a dimension level the mean incremental (AOTG vs control) and pre-post change controlled change in effect favoured AOTG in all dimensions, statistically significant at a 5% level of type I error for 10/13 if they had been considered partially. Key to robust coverage and comparability is triangulation for joint consideration across effects under uncertainty. Table 3 triangulates across joint dimension level evidence with simple (equal dimension and/or domain weighted) aggregation to estimate incremental (AOTG vs control) and pre-post change controlled range standardised domain and global effects (for a base case domain aggregation and sensitivity analysis direct dimension aggregation) and their bootstrapped distributions under uncertainty.

**Table 3:** AOTG vs Control pre-post change controlled and range std. mean domain and global effects with parametric bootstrapped 95% CIs

Simply triangulated (weighted) domains and global measures	AOTG vs control pre-post change controlled and range std. means (95% CI)
Global mental health	3.32 (1.31, 5.32)^*^
Resilience and preventative factors	5.75 (3.60, 7.89)^**^
Mental health literacy	9.92 (7.38, 12.44)^***^
Help seeking Intentions	4.55 (1.59, 7.52)^*^
Mental health and sport	4.35 (1.25, 7.45)^*^
Global measure (base case, equally weighted domains)	5.58 (4.19, 6.96)^***^
Global measure (sensitivity analysis equal dimension weights)	6.26 (4.86, 7.66)^***^

^*^statistically significant with < 1% type I error or *p* < 0.01

^**^statistically significant with < 0.1% type I error or *p* < 0.001

^***^statistically significant with < 0.001% type I error or *p* < 0.00001

In interpreting domain level incremental findings (Fig. 2), Table 3 shows that standardised differences as a percentage of their scales range are significant with a lower than 1% level of type I error for each domain, indeed less than 0.1% for resilience and preventative factors and less than 0.001% for mental health literacy. Aggregate global effect measures for a base case with equal weights for domains and dimensions within domains or sensitivity analysis with equal weights for all dimensions ignoring domains are also both significant at a < 0.001% level.

The very high levels of certainty of standardised domain and global incremental effect change benefits for AOTG relative to control reflect orders of magnitude less variability within and between arms than for their equivalent individual dimensions and that across dimensions in each case the mean incremental change between arms favoured AOTG, while 10/13 significantly at a 5% level. Hence, triangulation with joint aggregate consideration of effects is key, given partial consideration of a single effect would have not adequately covered effects, lead to greater uncertainty and effectively a lottery (10/13 chance) as to whether AOTG was considered effective depending on which dimension was considered.

Such triangulation is particularly key in assessing joint evidence from health promotion strategies [41] in community settings, and more generally in satisfying coverage conditions for robust health economic analysis across community and individual level strategies and interventions [7], as considered in discussion. While triangulated evidence makes clear the statistically significant incremental improvement across domains or globally and hence net clinical benefit with AOTG relative to usual care effects changes at a community level, to move towards net benefit consideration implementation costs of the AOTG program also need to be considered alongside potential for downstream costs impacts associated with effects.

### Resource use and cost results

The AOTG strategy was implemented across 8 clubs and 22 teams utilizing 6 AOTG staff as facilitators with the amount of staff time per club ranging from 10.5 to 35 h (mean 24.5 h), the time per team ranging from 5.25 to 14.5 h and the total staff time per participant ranging from 0.31 to 0.97 h. Including all on-costs, staff wages ranged from $40.74 to $47.11 per hour, depending on salary level. The mean hourly cost for staff members was $43.79 (*SD* = $3.49).

Club level mean cost was $1223.05 (*SD* = $478.26) and ranged from $556.79 to $1773.11. When attributed down to the team level, costs ranged from $278.39 to $779.13 per team with mean of $473.83 (*SD* = $162.91). When those costs were attributed down to an individual level and microcosted merchandise costs of $8.94 per participants were added, AOTG implementation cost per participant ranged from $24.85 to $64.43, with mean cost of $37.47 (*SD* = 10.44).

**Fig. 3:**
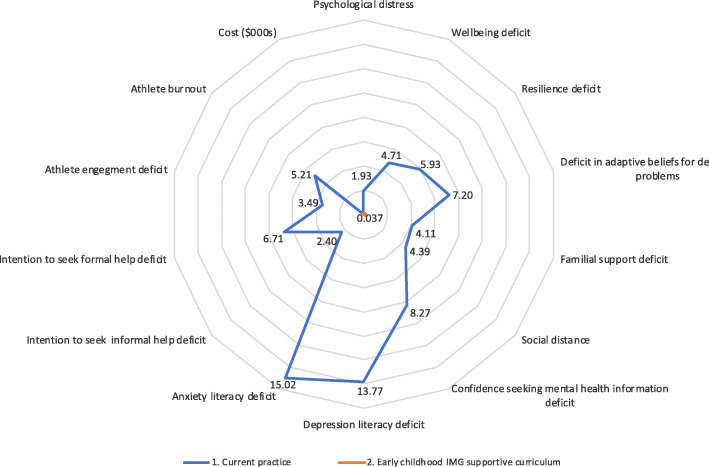
Incremental cost and 13 standardised incremental DU effects with flexible axes relative to lowest cost and lowest DU (highest effect) strategy

The mean AOTG cost per participant across 8 clubs and 22 teams of $37.47 were in turn attributable to club level costs (staff costs of club liaison and training and promotional materials) of $21.03 (range $8.05 to $45.35), team level costs (staff costs associated with presenting AOTG strategies to each team) of $7.49 (range $4.42 to $13.58) and participant level costs (merchandise cost) of $8.94, as shown in Table 4. Joint consideration of AOTG implementation costs alongside standardised effect differences for 13 domains are presented in Fig. 3.

**Table 4:** AOTG attributable implementation costs by team per participant for club, team and individual level (merchandise) costs

Club	Team	No. participants	Club level cost ($) / participant	Team level cost ($) / participant	Merchandise cost ($) / participant	Total cost ($)/ participant
1	1	24	14.38	5.89	8.94	29.21
1	2	24	14.38	5.89	8.94	29.21
1	3	14	14.38	10.10	8.94	33.41
1	4	22	14.38	6.42	8.94	29.74
2	5	18	8.06	7.85	8.94	24.85
2	6	18	8.06	7.85	8.94	24.85
2	7	15	8.06	9.42	8.94	26.42
2	8	17	8.06	8.31	8.94	25.32
3	9	12	45.30	10.19	8.94	64.43
3	10	17	45.30	7.19	8.94	61.43
4	11	9	25.64	13.58	8.94	48.17
4	12	14	25.64	8.73	8.94	43.32
4	13	13	25.64	9.40	8.94	43.99
5	14	18	31.87	3.40	8.94	44.21
5	15	14	31.87	4.37	8.94	45.18
6	16	16	18.87	8.83	8.94	36.64
6	17	17	18.87	8.31	8.94	36.13
6	18	18	18.87	7.85	8.94	35.66
6	19	13	18.87	10.87	8.94	38.68
7	20	14	34.72	5.05	8.94	48.71
8	21	8	31.68	4.42	8.94	45.03
8	22	8	31.68	4.42	8.94	45.03
	Overall	343	21.03	7.49	8.94	37.47

Interpreting Fig. 3 the AOTG program is shown to have the lowest expected DU and hence greatest benefit for each effect dimension or domain while direct implementation costs of the AOTG program are $37.47 per person.

### Sensitivity analysis with football only comparison AOTG vs control

Sensitivity analysis restricting participants to those in football clubs and teams removed 30 participants in the AOTG arm (14 from a swimming club and 16 from a NRL club) and 13 participants in the control arm (8 from a basketball club and 5 from an AFL club). Hence, a football (soccer) only comparison (Table 4) is based on 313 AOTG and 260 control arm participants.

Comparing football (soccer) only participants, each of the 13 effect dimensions remain in favour of AOTG over control with higher mean effect change or equivalently lower disutility, 11/13 statistically significant with 5% type I error and cost of implementing AOTG per participant reduced to $36.57 per participant (Table 5).

**Table 5:** Sensitivity analysis with football only participants and AOTG vs control raw and range standardised effects across 13 dimensions and implementation cost

Dimension	Raw score mean difference (95%CI)	Score Range	Standardised mean difference (95%CI)
Psychological distress	0.54 (−0.13, 1.20)	0–24	2.24 (−0.52, 5.01)
Wellbeing	3.81 (1.49, 6.12)^a^	10–84	5.14 (2.01, 8.27)^a^
Resilience	2.64 (1.28, 4.00)^a^	10–50	6.60 (3.20, 9.99)^a^
Adaptive beliefs for dealing with problems	1.06 (0.33, 1.79)^a^	3–18	7.08 (2.23, 11.93)^a^
Familial support	0.25 (0.02, 0.48)^a^	1–7	4.11 (0.25, 7.97)^a^
Social distance	0.19 (0.03, 0.35)^a^	1–5	4.75 (0.66, 8.84)^a^
Confidence seeking mental health information	0.22 (0.02, 0.43)^a^	1–5	5.62 (0.39, 10.85)^a^
Depression literacy	1.80 (1.27, 2.38)^a^	0–13	13.87 (9.39, 1836)^a^
Anxiety literacy	1.90 (1.27, 2.53)^a^	0–13	14.64 (9.80, 19.48)^a^
Intention to seek informal help	0.10 (−0.14, 0.34)	0–7	1.45 (−1.96,4.86)
Intention to seek formal help	0.46 (0.16, 0.76)^a^	0–7	6.57 (2.36, 10.79)^a^
Athlete engagement	0.19 (0.001, 0.37)^a^	1–5	4.62 (0.01, 9.24)^a^
Athlete burnout	0.25 (0.08, 0.43)^a^	1–5	6.31 (1.97, 10.66)^a^
Implementation cost	$36.57 ($35.40, $37.74)		

^a^statistically significant at 5% level type I error

Hence, sensitivity analysis restricting comparison between AOTG and usual practice to football only clubs, teams and participants further reinforces the findings of AOTG being both an effective and low cost mental health promotion strategy.

More generally, joint consideration of effects and their associated long term health system cost implications, whether in base case or sensitivity analysis in Fig. 3 and Tables 4 and 5, point to the potential for long term net cost savings if there were community ownership of AOTG as health promotion strategy, as considered in greater detail in discussion.

## Discussion

This paper has undertaken health economic multi-dimension cost effectiveness analysis in the third phase of the AOTG study evaluating the Ahead of the Game mental health promotion strategy for adolescence males and its implementation in practice in community sporting club settings developed in the first two phases [39, 40]. Methods have been developed to enable robust evaluation of the AOTG strategy and its combined implementation impacts in practice triangulating on evidence across 13 mental health dimensions, 5 domains and AOTG implementation costs.

Within study triangulated analysis of jointly estimated differences of effects under uncertainty has demonstrated that the AOTG strategy is associated with mental health improving across all 13 dimensions with 10/13 significantly at 5% level type 1 error, across all 5 mental health domains (less than 1% level type 1 error in each case) and with simply weighted overall aggregate measures from domain or dimension level (less than 0.001% level type 1 error in each case), while having conservatively estimated per participant expected implementation cost of $37.47.

Extrapolating beyond the within study analysis presented in this paper, AOTG would be net cost savings to the health system in the long term if the net present value of cost saving to the health system from downstream improved mental health effects were greater than $37.47 per participant. That is, from a long-term health system perspective integration of AOTG program into clubs would be expected to dominate current practice if the net present value of downstream cost savings associated with improved effects were $37.47 per individual or greater. If there is long term community ownership of AOTG community strategies the downstream potential cost savings from successful community level health promotion and disease prevention strategies such as AOTG are significant given community network and multiplier effects that are then expected to arise in increasing population scope and duration of effects [7, 9, 17, 19, 20, 34, 35].

That is, combined population improvements with AOTG across the mental health literacy domain by almost one tenth of its range (9.92/100), resilience and preventative factors by 5.75/100, health seeking intentions by 4.55/100, Mental health and sport by 4.35/100 and global health by 3.32/100 would be expected to lead to long term population level cost savings where the AOTG program had sports community (club, team and participant) ownership with policy and practice integration. The development of the AOTG strategy and model of implementation to optimise ownership, implementation and integration in phase one and two of the AOTG study alongside participant dimensions and domains of effect that reflect community ownership and help seeking intentions support such potential cost savings with AOTG.

Extending within study analysis in Fig. 3 to such longer-term consideration of cost and effect dominance of AOTG would be simply represented in C-DU space with incremental cost and each effect framed from a disutility (DU) perspective for AOTG being at the origin. Importantly then, the AOTG strategy has significant potential to be net cost saving as well as effective as a community health promotion strategy with scope for multiplier effects across community networks and with pricing at factor costs. This potential is precluded with individual level interventions with ‘value based’ pricing such as pharmaceuticals both because they are priced up to ‘value thresholds’ and as interventions targeted at individuals do not enable multiplier effects beyond that target population [7, 8, 29, 30].

Never-the-less, while beyond the scope of this paper, to better assess and model the potential for population level community level effect of community health promotion strategies such as AOTG and the extent of associated downstream health system cost savings quantitative analysis would ideally assess community multiplier and more generally network effects over time [7, 9, 20, 34]. Future research in that respect would be valuable in quantifying the extent of long-term effects and downstream cost savings with AOTG as with any community level health promotion strategy. Such research is more generally key to identifying effective and net cost saving community health promotion strategies that health systems should be prioritising for investment [33].

Overall, AOTG study findings demonstrate an inexpensive health promotion strategy which in addition to having expected benefits in promoting mental health having significant potential to be net cost saving to the health system relative to current practice with integration into sporting clubs and organisation supported by community ownership.

### Study strengths and weaknesses

One aspect of that study analysis that could be considered a limitation is a lack of consideration of multiple effect adjustments. However, any such conventional consideration of multiple effect adjustment only serves to reinforce the central finding of the study of the perils of partialisation with fragmented consideration of multiple effects and key importance of the robust methods illustrated for jointly considering effects. For example, with the most conservative conventional Bonferroni correction for multiple comparisons the appropriate *p* value with a one sided test for a global level is *p* < 0.05, while at a domain level it would be *p* < 0.01 = 0.05/5, and a single effect level *p* < 0.0076 = 0.05/13 or half that with a 2 sided test. Hence, undertaking multiple comparisons the overall global improvement with AOTG remains highly significant (*p* < 0.00001), while each of the domains remain significant with two or one sided tests. However at an individual effect level only 6 of 13 would remain significant with a multiple effect adjusted two sided test (wellbeing, resilience, dealing with problems, depression literacy, anxiety literacy) and 7/13 with a one sided test (athlete burnout also significant). Consequently, multiple effect comparison methods reinforce the perils of partialisation, making even clearer the lottery that conventional partial consideration of effects leads to and the need for their joint consideration.

More generally, robustly informing public sector decision making with health economic analysis requires evidence synthesis with joint consideration of decision analytic principles of coverage and comparability to avoid biases in estimating incremental effects and costs and consequently undertaking cost effectives or net benefit analysis [6].

Coverage alongside comparability is particularly key for evaluating health promotion strategies such as AOTG in community settings where triangulation across quantitative as well as qualitative evidence for individual and community (e.g. team, club and wider community) ownership, attitudes, lifestyle and behaviour factors are key to continuation of the program, expected individual effects, multiplier effects and subsequently the effectiveness and cost effectiveness of the strategy [7, 9].

In evaluating AOTG as a health promotion strategy triangulation across community level pre-post matched evidence for each of 5 domains and 13 dimensions were each important to assessing the success and net effect of the program. The extended NBCT methods presenting multiple effects with radar plots in C-DU space and bootstrapping methods for triangulating evidence have been illustrated to combine and summarise evidence in enabling robust joint consideration of AOTG multiple effects alongside implementation costs under uncertainty.

Importantly the adapted and extended NBCT methods enable informed decision making with intuitive joint comparison of multiple effects alongside costs consistent with maximising net benefit with natural improvement where cost or effects presented from a disutility perspective are minimised. By jointly presenting dimension level analysis such as that in Fig. 1b and c prior to any aggregated analysis such as in Fig. 2 and Table 3 under uncertainty the method also flexibly enables alternative weightings in aggregating to domain or global level to reflect preferences across effects of any given jurisdiction at any point in time. Hence, while triangulation to domain and global level in Fig. 2 and Table 3 a priori employed equal dimension and/or domain weights, if alternative weights reflecting preferences in any given jurisdiction across dimensions and or domains were known, they naturally can be simply applied.

In relation to comparability, for community level evidence of interest in evaluating health promotion strategies ideally randomising allocation to AOTG or usual care across many communities would be undertaken. Never the less as a prospective study of community level effects where trial funding limited the feasibility of randomising across communities, undertaking differences in differences analysis triangulating between pre-post and matched community level evidence represented the strongest feasible quantitative study design. Undertaking pre-post and matched analysis together addresses many of the weaknesses of undertaking each separately. Pre-post analysis i.e. controlling for baseline effects addresses limitations in controlling for population differences between arms. Undertaking matched analysis over the same timeframe addresses the weakness of pre-post analysis in not controlling for impacts of external environment beyond the study intervention across intervention communities e.g. legislative or peak sporting body policy changes arising across communities and their clubs over time. Naturally, further research randomising between the AOTG implementation strategy and other implementation strategies across multiple communities would be valuable to further improve comparability, but should not be at the expense of compromising coverage.

In relation to coverage, adapting and extending NBCT methods with radar plot presentation in C-DU space and triangulation under uncertainty with bootstrapping methods have been shown to facilitate robust joint consideration across multiple effect domains alongside AOTG implementation cost. Importantly radar plot presentation in C-DU space retains the key advantages of the NBCT in enabling radial comparison consistent with maximising net benefit [10–13, 25, 26] while extending multiple effect presentation and triangulated uncertainty analysis to more than 3 dimensions. Like previous multiple domain of effect health economic research methods [25, 26], health economic analysis undertaken in this paper extends deterministic cost consequences and single effect cost effectiveness categories to enable robust multiple domain cost effectiveness analysis.

In many complex community settings such as health promotion or palliative care key incremental effects cannot be integrated into a single effect such as patients’ quality-adjusted life years (QALYs) and hence health economic analysis to robustly inform decision making requires coverage of multiple effects alongside costs under uncertainty, to enable robust unbiased analysis [25–27]. For example, allowing for palliative patient primary domains of finalising affairs, carer and family effects and being in their community of choice with who they want to be with; each of which cannot be integrated with patient survival in patient QALY analysis [27]. Similarly, in community health promotion settings such as this study, multiple dimensions across mental health resilience and preventative factors, help seeking intentions, use of network support and community ownership and network impacts at a sporting organisation, club and team level cannot be integrated into a single effect measure.

Unlike previous applications of the NBCT to comparison of multiple effects with orthogonal presentation in cost-disutility space, comparison in cost disutility space for AOTG intervention relative to usual care across 13 effects and cost shown in this paper employ non-orthogonal methods. These non-orthogonal methods enable both simple and feasible joint presentation of the AOTG studies 13 effects and costs data not possible with orthogonal methods, and for deterministic analysis can graphically summarise all data in one diagram.

The non-orthogonal methods presented here are otherwise more constrained than orthogonal methods in scope of analysis for informing societal decision making allowing for joint effect and cost uncertainty and in extending to summary measures such as expected net loss curves, frontiers, planes and surfaces [1, 11, 12, 25, 26]. Hence, use of orthogonal methods to complement analysis presented in this paper are suggested for further research in extending associated summary measures. Never-the-less, the joint consideration of 13 effects and costs and hence 14-dimensional analysis in evaluating the AOTG strategy as a health promotion strategy extends dimensionality of multiple domain cost effectiveness analysis from three-dimensional analysis previously considered employing orthogonal NBCT methods in palliative settings.

Robust methods for undertaking multiple domain cost-effectiveness analysis are important to allow appropriate coverage in many areas where multiple effect domains cannot be collapsed into a single effect measure such as QALYs. Indeed, even for cases where QALYs enable a single effect measure integrating survival and quality of life aspects, the weights of multiple effects and dimension contributing to QALY measurement are rarely transparent and can be a black box for decision makers. Consequently, further research is also suggested as valuable in extending the multiple dimension evaluation methods presented here to enable transparent QALY analysis. That is, to allow explicit joint consideration of multiple dimensions and domains of effects in QALY analysis where weights can differ with different preference sets across jurisdictions but also over time.

## Conclusion

The methods identified for undertaking multiple effect comparison and cost effectiveness analysis for the AOTG study in this paper have adapted and extended robust net benefit correspondence theorem methods enabling joint presentation with radar plots of 13 effect dimensions alongside costs in C-DU space and their triangulation under uncertainty with robust bootstrapping methods. Employing these methods statistically significant benefits were found for AOTG effect changes incremental to usual care on 10/13 dimensions and all 5 domains (*p* < 0.01) or global measures (*p* < .0001) with simple dimension and/or domain aggregation. This analysis demonstrates AOTG effectiveness as a mental health promotion strategy and points to AOTG dominating current practice if down streaming cost savings associated with better mental health management are greater than the estimated $37.47 direct costs per individual of implementing the AOTG program. The multidimensional extension of net benefit correspondence theorem methods with radar plot presentation for their joint consideration in cost-disutility space, triangulation and bootstrapping methods developed and illustrated for the AOTG study have distinct advantages over cost-consequences analysis in flexibly allowing for joint cost and multiple effect dimensions and domains comparison and their aggregation and triangulation under uncertainty consistent with net benefit maximisation. This approach improves triangulation and coverage of multiple domains key for evaluation in settings such as health promotion without compromising comparability and avoid perils of partialisation that otherwise arise with multiple single effect cost effectiveness analyses.

## Data Availability

The datasets analysed during the current study are available from the corresponding author on reasonable request.

## Abbreviations

AOTG: Ahead of the Game (intervention and study)
C-DU space: Cost-disutility space
DU: Disutility (effect measure and effect framing)
NBCT: Net benefit correspondence theorem
QALY: Quality adjusted life year
